# A Moroccan Experience in the Management of Acute Coronary Syndrome Without ST-Segment Elevation: Clinical Profiles and Therapeutic Approaches

**DOI:** 10.7759/cureus.80155

**Published:** 2025-03-06

**Authors:** Soufiane Touiti, Sara El Guessabi, Kamal Marzouki, Amale Tazi Mezalek, Aatif Benyass

**Affiliations:** 1 Cardiology, Mohammed V University, Rabat, MAR; 2 Epidemiology and Public Health, Rabat Medical and Pharmacy School, Mohammed V University, Rabat, MAR; 3 Cardiology, Abulcasis International University of Health Sciences, Rabat, MAR; 4 Cardiology, Cheikh Zaid International University Hospital, Rabat, MAR; 5 Cardiology, Mohammed V Military Instruction Hospital, Mohammed V University, Rabat, MAR

**Keywords:** coronary artery disease, epidemiology, myocardial infarction, percutaneous coronary intervention, risk stratification

## Abstract

Objective

Acute coronary syndrome without ST-segment elevation, which includes Non-ST-segment elevation myocardial infarction (NSTEMI), represents a critical medical emergency due to its high morbidity and mortality rates, its economic impact on public health, and its steadily increasing incidence. This study aims to investigate the epidemiological, clinical, paraclinical, and therapeutic profiles of NSTEMI patients at Cheikh Zaid International University Hospital.

Methods

This retrospective, single-center study was conducted over a period of three years, from January 2020 to December 2022, at Cheikh Zaid International University Hospital. It included 140 patients who were admitted directly to the cardiology department or through the emergency department for NSTEMI, provided their medical records were complete and usable for clinical, paraclinical, therapeutic, and evolutionary data. NSTEMI was diagnosed based on a combination of clinical criteria, including chest pain, electrocardiogram (ECG) findings such as ST-segment depression or T-wave inversions, and elevated troponin levels above the established threshold (≥35 pg/mL).

Results

The mean age of the study population was 66±9.64 years. Males constituted 77% (n=108) of the cohort. The most common cardiovascular risk factors were hypertension (53.6%, n=75), diabetes (50.7%, n=71), and smoking (34.3%, n=48).

Biochemical markers showed elevated troponin levels (>35 pg/mL in 72.66%, n=101) and LDL-C levels >0.55 g/L (n=92, 65.7%). Echocardiography revealed hypokinesia in 49.3% (n=69) and moderate to severe left ventricular dysfunction (LVEF <50%) in 37.86% (n=53). Coronary angiography showed single-vessel disease in 43.6% (n=61), with the anterior interventricular artery affected in 44% (n=62).

Regarding treatment, 58.5% (n=82) underwent percutaneous coronary intervention (PCI), 27% (n=37) underwent coronary artery bypass grafting (CABG), and 15% (n=21) were managed with medical therapy alone. The in-hospital mortality rate was 3% (n=4). Beyond mortality, complications such as heart failure and recurrent ischemia were observed in a subset of patients, further emphasizing the need for timely intervention.

Conclusions

Delayed admission is associated with worsened patient outcomes by limiting the efficacy of reperfusion strategies. High-risk patients require urgent management in line with international guidelines. To improve survival and long-term prognosis, increasing public awareness, particularly about early symptom recognition, and improving pre-hospital triage infrastructure are essential. Further, enhancing the public’s understanding of cardiovascular risk factors and the importance of early medical consultation could significantly impact the reduction of delays in care.

## Introduction

Cardiovascular diseases are the leading cause of morbidity and mortality in industrialized countries and are increasingly becoming a significant concern in emerging nations. Acute coronary syndrome (ACS) is a critical component of this group of diseases and represents a major medical emergency that any physician may encounter during their practice [1].

In the United States, ACS affects approximately 1 million patients annually, with approximately 70% presenting as non-ST-segment elevation myocardial infarction (NSTEMI) [2,3]. Similarly, in Morocco, ACS poses a substantial public health challenge, with high incidence and mortality rates. However, unique challenges complicate the management of ACS in Morocco, including limited access to healthcare, delays in diagnosis, and variability in treatment availability across regions [4]. These factors contribute to poorer outcomes, underscoring the urgent need for improved diagnostic and therapeutic strategies to address the growing burden of ACS in the country.

The role of atherosclerosis in the development of coronary artery disease, and specifically in the occurrence of ACS, has been well established over the past few decades [5]. In Morocco, as in many other countries, chest pain affects a significant portion of the population annually, making it one of the most common presentations in emergency departments [6]. Accurate diagnosis of ACS relies on a comprehensive assessment, including patient history, physical examination, risk factor evaluation, electrocardiography, biomarker analysis, risk stratification, and, when necessary, coronary angiography. Given the specific challenges in Morocco, such as diagnostic delays and limited resources, a detailed understanding of local clinical and paraclinical characteristics is crucial.

The primary objective of this study is to delineate the epidemiological, clinical, and paraclinical profiles of NSTEMI patients managed at the Cheikh Zaid International University Hospital in Rabat. Additionally, the study aims to assess the therapeutic approaches used in this setting, evaluate patient outcomes, and compare these findings with international guidelines and existing literature. By identifying areas where local practices diverge from international standards, the study will formulate recommendations to enhance the management of NSTEMI at Cheikh Zaid International University Hospital and, more broadly, in Morocco.

## Materials and methods

Type of study

This is a retrospective, observational, descriptive, and analytical study conducted on 140 patients diagnosed with NSTEMI at the Cardiology Department of the Cheikh Zaid International University Hospital between January 2020 and December 2022. The study was approved by the Ethics Committee of the Abulcasis International University of Health Sciences (UIASS). This study is primarily descriptive and analytical, focusing on clinical, paraclinical, and therapeutic profiles of patients, and includes both cross-sectional and time-to-event analyses (e.g., time to coronary angiography, time to revascularization, and outcomes such as survival rates). Patients were selected consecutively based on the availability of complete medical records meeting the inclusion criteria. This approach minimized selection bias by ensuring that all eligible patients admitted to the hospital during the study period were considered for inclusion.

Population characteristics

The study included patients who were admitted either directly to the cardiology department or through the emergency department at Cheikh Zaid International University Hospital for NSTEMI, provided their medical records were complete and met predefined criteria for data inclusion, ensuring the availability of clinical, paraclinical, therapeutic, and evolutionary data. Specifically, complete records were defined as those containing sufficient information on sociodemographic characteristics, cardiovascular risk factors, clinical presentation, diagnostic test results (e.g., ECG, biomarkers), therapeutic interventions, and follow-up data. Patients were selected consecutively during the study period to minimize selection bias.

Patients with a history of previous myocardial infarction (MI) were included in the study. Their data were handled separately in the analysis to assess potential differences in outcomes based on prior MI, with special attention to how this factor might influence treatment approaches and prognosis. NSTEMI was diagnosed based on a combination of clinical presentation (e.g., chest pain, dyspnea), electrocardiographic (ECG) findings (absence of ST-segment elevation but possible ST-depression or T-wave inversion), and specific biomarker thresholds. The diagnosis required elevated cardiac biomarkers (e.g., troponin I or T) exceeding the 99th percentile of the upper reference limit, with corresponding ECG and clinical features confirming the suspicion of myocardial ischemia.

Data collection

Data were collected retrospectively from patients' medical records using a standardized form that included all relevant and available parameters. The form encompassed sociodemographic characteristics and medical history, including cardiovascular risk factors such as hypertension, diabetes, smoking, dyslipidemia, family history of coronary disease, menopause, and prior coronary or vascular events. Clinical data recorded at admission included presenting symptoms (e.g., chest pain, dyspnea), blood pressure, respiratory rate, heart rate, temperature, and findings from cardiovascular and systemic examinations.

Paraclinical data included electrocardiographic (ECG) findings, biological markers such as cardiac enzymes, coagulation profile, renal function, blood electrolytes, blood glucose, lipid profile, and CRP, along with echocardiographic and coronary angiographic findings. Risk stratification was performed based on ischemic risk, the Global Registry of Acute Coronary Events (GRACE) score, hemorrhagic risk, and the time elapsed between admission and coronary angiography. The GRACE score was calculated retrospectively from the patient records and used to categorize patients into very high, high, or low-risk groups. This score incorporates eight parameters: age, systolic blood pressure, heart rate, creatinine levels, ST-segment deviation, elevation of cardiac markers, cardiac arrest, and Killip class.

In cases where coronary angiography findings were available, the severity of coronary lesions was considered as part of the risk stratification, and angiographic severity scores (e.g., SYNTAX score) were also evaluated, if available, to further refine the risk assessment. The therapeutic strategy documented included medical treatment and revascularization approaches, either percutaneous coronary intervention (PCI) or surgery.

Follow-up data for post-discharge outcomes were obtained through structured follow-up visits, phone calls, and review of medical records during the study period. These data included short-term, medium-term, and long-term outcomes such as readmissions, complications, and mortality. All collected data were entered into Excel 2021 for statistical analysis.

Biostatistical analysis

Data analysis was performed using JAMOVI software (Version 2.3.21.0). Qualitative variables were expressed as counts and percentages and analyzed using the chi-squared test or Fisher's exact test, depending on the conditions of application. Quantitative variables with a symmetrical distribution were presented as mean±standard deviation, while those with an asymmetrical distribution were expressed as median and quartiles. Comparisons of quantitative variables between two independent samples were conducted using the student’s t-test for symmetrical distributions or the Mann-Whitney U test for asymmetrical distributions. Statistical significance between compared groups was determined with a p-value threshold set at <0.05.

## Results

Epidemiological data

The 140 patients included in our study were predominantly male, with 108 men (77%) and 32 women (23%), resulting in a male-to-female ratio of 3.4:1. This ratio is consistent with findings in the existing literature, where male patients often outnumber female patients in NSTEMI cases. However, a comparison with regional or national statistics on NSTEMI could provide further context on gender distribution in our setting. The average age of the study population was 66±9.64 years, with the youngest patient aged 43 years and the oldest 87 years. The mean age for male patients was 65.4±9.17 years, while for female patients, it was 68±11 years. The most represented age group was 60-70 years, with 53 patients (38%).

Among the total study population, 127 patients (90.7%) were from urban areas, while 13 patients (9.3%) resided in rural areas. No significant differences were found in the prevalence of risk factors or outcomes between these two groups, although a larger sample size could provide more insight into potential disparities in rural versus urban populations. The cardiovascular risk factors assessed included diabetes, hypertension, smoking, dyslipidemia, obesity, coronary heredity, and menopause in women. The most prevalent risk factors were hypertension (75 patients, 53.6%) and diabetes (71 patients, 50.7%), followed by chronic smoking (48 patients, 34.3%). Dyslipidemia was found in 38 patients (27.1%), and coronary heredity was noted in 9 patients (6.4%). Obesity, defined by a BMI≥30 kg/m², was present in 20 patients (14.3%). Coronary heredity was diagnosed based on a family history of early cardiovascular events. Regarding menopause, 23 women (16.4%) were included, but specific details on the age at menopause (whether natural or surgical) were not collected, which may influence risk stratification and treatment outcomes.

The primary comorbidities identified included a history of ischemic heart disease in 40 patients (28.6%), stroke in 12 patients (8.6%), atrial fibrillation in 5 patients (3.6%), and chronic kidney disease in 5 patients (3.6%). Peripheral arterial disease of the lower limbs was documented in only 1 patient (0.7%). The presence of atrial fibrillation and peripheral arterial disease could impact patient outcomes and was taken into consideration in the prognostic assessments (Table 1).

**Table 1:** General characteristics of patients (N=140). Data represented as N (%) or Mean±SD. p-value is considered significant at p<0.05.

Variables	N	%
Sex		
Male	108	77
Female	32	23
Age		
Mean±SD	66±9.64	
<50	6	4
50-60	30	21
60-70	53	38
70-80	39	28
>80	12	9
Geographic origin		
Urban	127	90.7
Rural	13	9.3
Risk factors		
Hypertension	75	53.6
Diabetes	71	50.7
Smoking	48	34.3
Dyslipidemia	38	27.1
Coronary heredity	9	6.4
Obesity	20	14.3
Menopause	23	16.4
Comorbidities		
Stroke	12	8.6
Chronic kidney disease	5	3.6
Atrial fibrillation	5	3.6
Peripheral arterial disease	1	0.7

Clinical presentation

Symptoms and Consultation Reasons

Chest pain was the most common reason for consultation, occurring in 134 patients (95.7%). Among those presenting with chest pain, 41 patients (29.3%) had resting angina, 37 patients (26.4%) experienced crescendo angina, and 33 patients (23.6%) reported de novo angina. Resting angina was characterized by chest pain occurring at rest or with minimal exertion, crescendo angina involved increasing severity and frequency of chest pain over time, and de novo angina referred to chest pain that occurred for the first time in a patient's clinical history. Additionally, 2 patients (1.4%) presented with atypical angina, which was defined as chest pain with non-classical features, such as pain not associated with exertion or without the typical radiation or duration of pain.

A delay in admission was noted, with half of the patients (70 patients) being admitted 2 days after the onset of symptoms. A quarter (35 patients) were admitted within 1 day. The median time to admission was recorded, though a range of delays was observed. In terms of associated symptoms, 40 patients reported dyspnea, syncope, and palpitations during the medical history interview.

Physical Examination

At the clinical examination, 12 patients (8.6%) presented signs of left heart failure, 4 patients (2.9%) had signs of right heart failure, and 4 patients (2.9%) had evidence of peripheral artery disease. Cardiac murmurs were noted in 8 patients (5.7%), while 3 patients (2.1%) exhibited irregular heart sounds upon auscultation. However, 117 patients (83.6%) showed no abnormalities on physical examination (Table 2).

**Table 2:** Clinical characteristics of patients. Data represented as N (%).  p-values were calculated using the chi-square test (p<0.05 is considered significant).

Variables	N	%
Reason for consultation		
De novo angina	33	23.6
Crescendo angina	37	26.4
Resting angina	41	29.3
Atypical angina	2	1.4
Dyspnea	34	24.3
Syncope	4	2.9
Palpitations	2	1.4
Physical Signs		
Signs of left heart failure	12	8.6
Signs of right heart failure	4	2.9
Cardiac murmur	8	5.7
Vascular abnormalities	4	2.9
Irregular heart sounds	3	2.1
Normal exam	117	83.6

Electrocardiogram (ECG) Findings

ECG abnormalities were detected in 119 patients (85%), with ischemic signs such as ST-segment depression in 27 patients (19.2%), repolarization abnormalities in 75 patients (53.3%), pathological Q waves in 22 patients (15.8%), and R wave attenuation in 18 patients (12.5%). The repolarization abnormalities were further categorized, including T-wave inversions and ST-segment changes, which are commonly seen in NSTEMI cases and are indicative of ischemia or myocardial injury.

Interestingly, some patients had completely normal ECGs, which highlights the limitations of ECG in diagnosing NSTEMI in certain cases. The presence of R wave attenuation is clinically significant as it can indicate myocardial ischemia, typically in the anterior or lateral wall, and could potentially guide therapeutic decisions, such as the urgency of revascularization. Additional findings included complete left bundle branch block (LBBB) in 27 patients (19.2%), left ventricular hypertrophy (LVH) in 5 patients (3.3%), and atrial fibrillation (AF) in 5 patients (3.3%).

Ischemic changes were primarily observed in the anterior territory, with 43 patients (30.7%) showing changes in the anteroseptal region, 33 patients (23.6%) in the anterior region, and 52 patients (37.1%) in the lateral region. Ischemic involvement of the anterior wall is particularly concerning as it is associated with a higher risk of heart failure, arrhythmias, and adverse outcomes, necessitating closer monitoring and potentially more aggressive treatment strategies. In contrast, the inferior region was affected in 46 patients (32.9%), but ischemia here is often less likely to result in significant left ventricular dysfunction compared to anterior ischemia. Basal territory involvement was minimal, with only 1 patient (0.7%), and no abnormalities were found in the right territory, which is typically less relevant in the context of NSTEMI.

Biological Findings

High-sensitivity Troponin I (H0) was measured in 139 patients, with positive results in 101 cases (72.66%) and negative results in 38 cases (27.34%). The median troponin value was 237.54 pg/mL (IQR: 36-1144 pg/mL), ranging from a minimum of 36 pg/mL to a maximum of 50,000 pg/mL, with a positive threshold set at >35 pg/mL.

Troponin kinetics at H3 were only assessed in 24 patients. This subset was selected based on clinical judgment, considering the severity of symptoms, initial troponin levels, and the need for further risk stratification. The decision to perform H3 troponin testing was primarily driven by the clinical assessment of patients suspected of having ongoing myocardial injury. Of these, 14 patients had positive results consistent with NSTEMI, while 10 had negative results, suggesting unstable angina. The small number of patients in this group limits generalizability, but the troponin kinetics in these individuals were used to better understand the timing of myocardial injury and refine risk assessment.

Troponin levels, particularly the kinetics observed at H0 and H3, were found to correlate with both the ECG findings and clinical severity. Elevated troponin levels are often associated with more extensive ischemia or infarction, which is supported by ECG findings such as ST-segment depression and repolarization abnormalities. Higher troponin levels are also correlated with more severe clinical presentations, including signs of heart failure and worse outcomes, reinforcing the importance of integrating biomarker data with clinical and ECG findings.

Echocardiographic Data

Echocardiographic evaluation revealed that 69 patients (49.3%) exhibited wall motion abnormalities, with 18 patients (12.9%) showing akinesia and 58 patients (41.4%) having normal wall motion. These abnormalities are important indicators of myocardial injury and are closely associated with poor prognosis and a higher risk of complications such as heart failure and arrhythmias. Left ventricular ejection fraction (LVEF) is a critical marker in NSTEMI patients; 19 patients (13.6%) had LVEF<40%, 34 patients (24.3%) had LVEF between 40-50%, and 87 patients (62.1%) had LVEF>50%. Reduced LVEF is indicative of more significant myocardial damage and can directly impact ischemic risk, as lower LVEF is associated with a higher risk of adverse cardiovascular events and poorer outcomes. Left atrial dilation (>40 mm) was found in 24 patients (17.1%), which could indicate chronic volume overload, typically due to left ventricular dysfunction. This is an important consideration in risk stratification, as patients with left atrial dilation are at higher risk for thromboembolic events, including atrial fibrillation, which was noted in 5 patients (3.6%) in our study. LV hypertrophy (LVH) was identified in 14 patients (10%), and it is important to note that LVH is often associated with a higher risk of arrhythmias, ischemic events, and worsened prognosis. Additionally, 13 patients (9.4%) had elevated LV filling pressures, which is commonly seen in those with left heart failure or diastolic dysfunction, though the latter was not specifically assessed in this cohort. The presence of elevated LV filling pressures further underlines the risk of heart failure in these patients (Table 3).

**Table 3:** Echocardiographic data. Data is represented as N (%). p-values were calculated using the chi-square test (p<0.05 is considered significant).

Variables	N	%
LVEF (%)		
<40%	19	13.57
40-50%	34	24.29
>50%	87	62.14
LV wall motion abnormalities		
Hypokinesia	69	49.3
Akinesia	18	12.9
Normal	58	41.4
Localization		
Anterolateral	28	20
Anteroseptal	38	27.1
Anterior	25	17.9
Apex	23	16.4
Inferoseptal	28	20
Inferolateral	27	19.3
Inferior	22	15.7

Risk Stratification

Ischemic risk stratification is crucial in guiding therapeutic decisions. In our study, ischemic risk was categorized as low in 24 patients (17%), high in 99 patients (70.7%), and very high in 17 patients (12.1%). This classification was based on clinical presentation, ECG findings, biomarkers (including troponin levels), and echocardiographic findings (e.g., wall motion abnormalities and LVEF). The GRACE score was applied to assess prognosis, with 21 patients (15%) having a score <100, 89 patients (63.6%) having a score between 100-140, and 30 patients (21.4%) having a score >140. The GRACE score is a well-established tool that incorporates several parameters, such as age, heart rate, systolic blood pressure, and biomarkers, to predict the risk of mortality and major adverse cardiovascular events (MACE) in NSTEMI patients. Higher GRACE scores are associated with poorer prognosis and guide decisions on more aggressive interventions, such as early PCI. Hemorrhagic risk was also evaluated using the HBR score. In our cohort, 125 patients (89.3%) had a low hemorrhagic risk, while 10 patients (7.1%) had a high risk, and 5 patients (3.6%) had a very high hemorrhagic risk. Hemorrhagic risk is an important factor when considering treatment options, especially anticoagulation therapies. Patients with high or very high hemorrhagic risk may require modified treatment strategies, such as the use of lower-dose anticoagulants or a preference for PCI over thrombolysis, to minimize the potential for bleeding complications (Table 4).

**Table 4:** Ischemic risk vs hemorrhagic risk. The data represented as N (%). ANOVA was used to determine statistical significance (p<0.05 significant).

Variables	N	%
Ischemic risk stratification		
Low ischemic risk	24	17
High ischemic risk	99	70.71
Very high ischemic risk	17	12.14
GRACE score		
<100	21	15
100-140	89	63.6
>140	30	21.4
Hemorrhagic risk		
Low	125	89.3
High	10	7.1
Very high	5	3.6

Coronary Angiography

Coronary angiography was performed on all patients, revealing single-vessel disease in 61 patients (43.6%), two-vessel disease in 27 patients (19.3%), and three-vessel disease in 42 patients (30%). Additionally, 10 patients (7.1%) were found to have non-significant lesions. For these patients with non-significant lesions, alternative explanations for their symptoms should be considered, such as microvascular disease or coronary artery spasm. These conditions can cause chest pain despite the absence of significant epicardial lesions and may contribute to the ischemic risk in these patients.

Lesion location was an important factor in ischemic risk stratification. Proximal lesions in the left anterior descending artery (LAD) and left circumflex artery (LCX) were particularly associated with higher ischemic risk, reflecting their impact on major myocardial territories. Moreover, the location of coronary lesions also correlated with elevated troponin levels, which were used to gauge the extent of myocardial injury. Early identification of high-risk lesions can guide timely revascularization strategies and help prioritize the most critical cases (Table 5). 

**Table 5:** Angiographic data. The data is represented as N (%). p-values were calculated using the Chi-square test (p<0.05 considered significant). LAD: left anterior descending artery; LCX: left circumflex artery, RCA: right coronary artery.

Variables	N	%
Coronary lesion location		
Left main trunk	7	5
Proximal LAD	50	35.7
Mid LAD	43	30.7
Distal LAD	7	5
Proximal LCX	21	15
Mid LCX	20	14.3
Distal LCX	5	3.6
Proximal RCA	29	20.7
Mid RCA	34	24.3
Distal RCA	12	8.6
Marginal branch	28	20
Diagonal branch	14	10
Bifurcation	1	0.7

The timing of coronary angiography in this study had a significant impact on patient outcomes. The mean time to angiography was 18 hours, with half of the patients undergoing the procedure within 12 hours. This timing was primarily guided by a combination of clinical risk stratification and logistical factors. High-risk patients, identified by biomarkers, ECG changes, and clinical presentation, were prioritized for early angiography, while lower-risk patients could undergo the procedure at a later stage. The timing of angiography is a key determinant of outcomes, as earlier intervention may improve survival rates, reduce the risk of complications, and increase the likelihood of successful revascularization.

Treatment

Pre-treatment

Pre-treatment was initiated in 44 patients (31.4%) using high doses of antiplatelet agents, notably clopidogrel combined with aspirin, along with intravenous bolus anticoagulation. Gastric protection was administered to all these patients. The pre-treatment approach aimed to stabilize the plaque, minimize thrombus formation, and prepare the patient for revascularization.

Revascularization

A total of 82 patients (58.57%) underwent balloon angioplasty with stent placement, while 37 patients (26.43%) received coronary artery bypass grafting (CABG). The decision between angioplasty, CABG, or medical therapy was based on lesion severity, patient comorbidities, and clinical judgment. For patients with isolated, high-grade lesions, angioplasty with stent placement was preferred, while CABG was reserved for those with multivessel disease or significant comorbidities that warranted more extensive intervention. For the remaining 15% of patients, medical therapy alone was used, either because they were not candidates for revascularization due to poor clinical status or because they had non-significant lesions.

Medical Treatment

Regarding discharge medical treatment, all patients (136) received medical therapy except for 4 patients who died during hospitalization. All patients received aspirin and a statin, and 93.4% were treated with a P2Y12 receptor inhibitor (83.1% on clopidogrel, 10.3% on ticagrelor). Gastric protection was given to 92 patients (67.6%). For anti-ischemic treatments, heart rate control was managed with beta-blockers in 102 patients (75%), while cardiac remodeling and blood pressure control were managed with renin-angiotensin-aldosterone system inhibitors in 87 patients (64%). For other medications, 24 patients (17.6%) received insulin and 7 patients (5.1%) oral antidiabetics for diabetes control. Additionally, 29 patients (21.3%) received calcium channel blockers for hypertension, and 7 patients were prescribed anticoagulants (VKAs or DOACs) due to atrial fibrillation.

Evolution

The average length of hospitalization was 4 days, with a minimum of 1 day and a maximum of 30 days. At least 50% of patients had a hospitalization of less than 3 days. The overall in-hospital mortality rate was 3%, with 4 patients dying during hospitalization. The majority of these patients were in the high ischemic risk group, as indicated by their clinical presentation, troponin levels, and ECG changes. In-hospital complications, such as acute renal failure (10 patients, 7.14%) and heart failure (6 patients, 4.29%), contributed to prolonged hospital stays and higher mortality rates. Follow-up data on patient outcomes post-discharge is currently being collected, but the majority of patients showed stable recovery with ongoing medical therapy.

Analytical statistics

A chi-square test (χ²) was used for categorical variables, a Student's t-test (t) for continuous variables, and ANOVA (F) for comparisons between multiple groups. The analysis revealed a significant association between smoking and gender (χ²=14.5, p<0.001) and obesity and gender (χ²=9.75, p=0.002). Additionally, troponin levels were significantly linked to mortality risk (F=11.2, p=0.004). Admission delay was also associated with increased mortality (F=15.2, p=0.003), while coronary angiography delay correlated with ischemic risk stratification (F=7.22, p=0.027). Length of hospital stay was significantly affected by diabetes history (t=1978.00, p=0.046) and multiple coronary lesions (F=11.3, p=0.01).

**Table 6:** Analytical statistics. *p-values were calculated using a chi-square test (χ²) for categorical variables, a Student's t-test (t) for continuous variables, and ANOVA (F) for comparisons between multiple groups.

Analysis variables	Test statistics (t/χ²/F)*	p-value
Smoking and gender	χ² = 14.5	
Obesity and gender	χ² = 9.75	0.002
Troponin and mortality risk	F = 11.2	0.004
Admission delay and mortality	F = 15.2	0.003
Coronary angiography delay and ischemic risk	F = 7.22	0.027
Length of stay with diabetes history	t = 1978.00	0.046
Length of stay with multiple coronary lesions	F = 11.3	0.01

## Discussion

NSTEMI is a prevalent and serious condition that significantly impacts patient prognosis. Its incidence continues to rise, primarily due to increasing life expectancy and an aging population. This study, to the best of our knowledge, represents the first Moroccan registry dedicated to NSTEMI patients and aims to address the following key points: Are Moroccan patients different from those in other countries? And does NSTEMI management in Morocco align with current international clinical guidelines?

In response to the first point, our cohort should be compared to international and national series. Epidemiological data are similar, indeed the average age of our patients was 66±9.64 years, closely aligning with findings from studies conducted in the ACVC-EAPCI EORP NSTEMI registry of the European Society of Cardiology (66 years) [7] and in local studies Agadir (61.6 years) [8] and Fès (63 years) [9]. Our cohort was predominantly male (77%), consistent with studies by Sylvain [10], Belle et al. [11], and the ACCESS study [12], as well as in the other Moroccan series. Additionally, the majority of our study population resided in urban areas, reflecting findings from the literature, which emphasize the difficulties rural patients face in accessing timely medical care.

In our cohort, the most prevalent cardiovascular risk factors were hypertension (53.6%), followed by diabetes (50.7%), and smoking (34.3%). These values are comparable to those reported in both Moroccan and European studies [13]. Hypertension and diabetes are well established as key contributors to atherosclerotic plaque development, facilitating plaque ulceration or rupture and thereby increasing the risk of ischemic heart disease.

One of the most concerning findings in our study was the significant delay between symptom onset and first medical contact in Morocco, which is substantially longer than in Europe, especially in the ACVC-EAPCI EORP NSTEMI registry of the European Society of Cardiology (6 hours) [7]. In our cohort, the average delay before seeking medical consultation was 48 hours. This prolonged delay directly impacts diagnosis and treatment, contributing to increased morbidity and mortality. These findings underscore the urgent need for healthcare professionals to raise awareness and educate patients on the importance of seeking immediate medical attention for chest pain.

Chest pain was the most commonly reported symptom, occurring in 95.7% of patients, in line with findings from comparative studies. Electrocardiographic (ECG) data revealed ST-segment depression in 19.2% of cases, a rate similar to that reported by Sylvain (24%) [10]. However, higher rates were observed in studies by Belle et al. (53.47%) [11] and at Agadir Hospital (61.4%) [8]. Additional ECG abnormalities included negative T waves in 47.5% of patients, consistent with findings from the Fès (46%) [9] and Sylvain studies (36%) [10]. Normal ECG findings were recorded in 14.3% of cases. These variations highlight the diverse clinical presentations of NSTEMI and reinforce the necessity of repeat ECGs for patients with persistent chest pain.

Regarding laboratory investigations, elevated troponin levels were detected in 72.66% of patients, a rate comparable to those reported by Belle et al. (73.5%) [11]. However, other studies have reported lower troponin positivity rates, ranging from 53% to 64%. Additionally, LDL-C levels in our cohort were notably higher than those reported in studies from Marrakech (25.6%) and Fès (48%) [9-15]. This discrepancy may be attributed to the increasing adoption of a Westernized lifestyle among Moroccan patients and the stricter 2023 ESC guidelines, which set an LDL-C target of 0.55 g/L for patients at very high cardiovascular risk [15].

Echocardiographic examination revealed that 58.6% of patients had impaired left ventricular function, a rate higher than in other studies. The left ventricular ejection fraction (LVEF) findings in our study closely aligned with those from the Marrakech study [15]. This may be explained by delayed NSTEMI diagnosis, which compromises coronary blood flow and subsequently impairs myocardial contractility.

Risk stratification is a critical step in guiding management decisions. In our study, 70.1% of patients were classified as high ischemic risk, a rate similar to that reported in several local series. However, only 12.14% of patients were categorized as very high ischemic risk, a lower proportion than in European studies.

Coronary angiography findings in our cohort revealed that monotruncal disease, particularly affecting the left anterior descending artery (LAD), was the most frequently observed abnormality, consistent with international studies. The distribution of affected coronary arteries in our study mirrored findings from the 2020 Fès series [9].

In terms of therapeutic management, 58.57% of patients underwent percutaneous coronary intervention (PCI) with stent placement, while 26.43% underwent coronary artery bypass grafting (CABG). Higher PCI rates (80-96%) have been reported in studies by Sylvain, Belle et al. [10,11], whereas lower rates (27-32%) were observed in Fès and Marrakech [9-15]. The proportion of CABG cases in our study closely matched findings from and Sylvain study [10].

Regarding pharmacological treatment, all patients received aspirin and statins, while 93.4% were prescribed P2Y12 receptor inhibitors in accordance with international guidelines. These results align with findings from national hospitals and studies conducted by Nadarajah et al., Sylvain, and Belle et al. [7,10,11]. For anti-ischemic therapy, beta-blockers and ACE inhibitors/ARBs were prescribed to 75% and 64% of patients, respectively, consistent with data from the Marrakech series [15].

The hospital setting, whether public or private, urban or rural, may greatly affect the applicability of our findings to the broader Moroccan population. Variations in healthcare infrastructure across regions can influence the management and outcomes of NSTEMI. In rural areas, patients often face delays in accessing timely medical care due to logistical challenges, under-resourced facilities, and lack of immediate healthcare staff. Moreover, recall and selection biases are valid concerns in retrospective studies. The timing of symptom onset, treatment choices, and other clinical factors may be particularly affected by these biases. Future studies should account for these biases and aim for more robust data collection methods, including prospective case-control or cohort studies. The availability of national registries could also play a pivotal role in facilitating long-term data collection, enabling us to refine our understanding of NSTEMI management in Morocco.

To answer the second point, the evaluation of our facility’s management strategies revealed that the therapies, revascularization techniques, and treatment timelines are in line with international standards. However, to further refine patient care and follow-up, several key improvements should be considered. First, the implementation of a structured information, education, and communication strategy, particularly targeting high cardiovascular risk patients, could significantly reduce delays in admission and treatment. Additionally, increasing awareness among individuals with chronic conditions such as hypertension and diabetes about the importance of regular medical follow-ups would aid in both the prevention and control of these risk factors, ensuring continuous therapeutic monitoring. Establishing a specialized center dedicated to the comprehensive management of these patients, equipped with a multidisciplinary team, would further enhance the quality of care. Finally, simplifying administrative admission procedures for urgent cases is essential to ensuring timely and efficient patient management. Collectively, these measures aim to optimize the healthcare pathway and improve patient outcomes.

Limitations

This study provides valuable insights into the epidemiological, clinical, and therapeutic management of NSTEMI in Morocco; however, several limitations must be acknowledged. First, as a single-center study, its findings may not be fully generalizable to other populations or healthcare settings. Additionally, its retrospective design introduces potential selection and recall bias as it relies on past medical records. Another limitation is the lack of long-term follow-up, as the study only captures in-hospital outcomes, preventing an assessment of long-term prognoses and complications. Moreover, incomplete data in some medical records may have affected the statistical robustness of our findings. Finally, the absence of a control group limits the ability to compare different management strategies or assess variations in outcomes between treated patients and a healthy cohort. These limitations highlight the need for larger, multicenter, and prospective studies to further refine our understanding of NSTEMI management in Morocco.

## Conclusions

Acute coronary syndrome is a major public health issue due to its frequency and severity, and NSTEMI remains a significant cause of cardiovascular morbidity and mortality. This study highlights the importance of early recognition, timely intervention, and adherence to evidence-based management strategies to improve patient outcomes. Our findings emphasize the role of hypertension, diabetes, and smoking as primary risk factors. Delayed admission correlates with worse prognoses, underscoring the need for enhanced public health initiatives. Prehospital delays may stem from a combination of factors, including patient hesitancy, inefficiencies in emergency response systems, and limited hospital accessibility. Addressing these issues in Morocco will require overcoming barriers such as resource limitations and healthcare disparities. Initiatives aimed at reducing these delays could focus on improving patient education, enhancing physician training, and implementing structural healthcare improvements within the system. However, practical challenges, such as limited resources and regional healthcare disparities, hinder the full implementation of these strategies.

Long-term prognostic studies are crucial for better understanding the outcomes of NSTEMI patients. Key outcomes of interest should include major adverse cardiovascular events, medication adherence, and quality of life. The development of national or multicenter registries could play an essential role in addressing research gaps and facilitating the collection of comprehensive data to inform future interventions. To optimize NSTEMI outcomes in Morocco, it is crucial to develop pre-hospital care systems, integrate early invasive strategies, and promote awareness campaigns about cardiovascular risk factors. Future research should explore long-term prognostic outcomes and the impact of different therapeutic interventions on morbidity and mortality, as well as assess how optimized risk stratification for intervention can further improve patient outcomes.
